# Optimizing combination therapy in prostate cancer: mechanistic insights into the synergistic effects of Paclitaxel and Sulforaphane-induced apoptosis

**DOI:** 10.1186/s12860-024-00501-z

**Published:** 2024-03-04

**Authors:** Tito N. Habib, Mohammed O. Altonsy, Salah A. Ghanem, Mohamed S. Salama, Mai A. Hosny

**Affiliations:** 1 Molecular Genetics Lab, Department of Zoology, Faculty of Science, Sohag University, Sohag, Egypt; 2 Snyder Institute for Chronic Diseases, University of Calgary, Calgary, AB T2N 4N1 Canada; 3 Department of Zoology, Faculty of Science, Ain Shams University, Cairo, Egypt

**Keywords:** Sulforaphane, Paclitaxel, Prostate Cancer, Cell cycle, Apoptosis, Combination therapy, Drug synergy

## Abstract

**Background:**

Combination therapies in cancer treatment have demonstrated synergistic or additive outcomes while also reducing the development of drug resistance compared to monotherapy. This study explores the potential of combining the chemotherapeutic agent Paclitaxel (PTX) with Sulforaphane (SFN), a natural compound primarily found in cruciferous vegetables, to enhance treatment efficacy in prostate cancer.

**Methods:**

Two prostate cancer cell lines, PC-3 and LNCaP, were treated with varying concentrations of PTX, SFN, and their combination. Cell viability was assessed using the thiazolyl blue tetrazolium bromide (MTT) assay to determine the EC50 values. Western blot analysis was conducted to evaluate the expression of Bax, Bcl2, and Caspase-3 activation proteins in response to individual and combined treatments of PTX and SFN. Fluorescent microscopy was employed to observe morphological changes indicative of apoptotic stress in cell nuclei. Flow cytometry analysis was utilized to assess alterations in cell cycle phases, such as redistribution and arrest. Statistical analyses, including Student’s t-tests and one-way analysis of variance with Tukey’s correction, were performed to determine significant differences between mono- and combination treatments.

**Results:**

The impact of PTX, SFN, and their combination on cell viability reduction was evaluated in a dose-dependent manner. The combined treatment enhanced PTX’s effects and decreased the EC50 values of both drugs compared to individual treatments. PTX and SFN treatments differentially regulated the expression of Bax and Bcl2 proteins in PC-3 and LNCaP cell lines, favoring apoptosis over cell survival. Our data indicated that combination therapy significantly increased Bax protein expression and the Bax/Bcl2 ratio compared to PTX or SFN alone. Flow cytometry analysis revealed alterations in cell cycle phases, including S-phase arrest and an increased population of apoptotic cells. Notably, the combination treatments did not have a discernible impact on necrotic cells. Signs of apoptotic cell death were confirmed through Caspase-3 cleavage, and morphological changes in cell nuclei were assessed *via* western blot and fluorescent microscopy.

**Conclusion:**

This combination therapy of PTX and SFN has the potential to improve prostate cancer treatment by minimizing side effects while maintaining efficacy. Mechanistic investigations revealed that SFN enhances PTX efficacy by promoting apoptosis, activating caspase-3, inducing nuclear morphology changes, modulating the cell cycle, and altering Bax and Bcl2 protein expression. These findings offer valuable insights into the synergistic effects of PTX and SFN, supporting the optimization of combination therapy and providing efficient therapeutic strategies in preclinical research.

**Supplementary Information:**

The online version contains supplementary material available at 10.1186/s12860-024-00501-z.

## Background

Combination therapy is a recommended intervention in which the patient receives more than one therapy. Treatment regimens involving the administration of several separate pills, each containing a specific drug, or single pills containing different drugs are examples of combination therapy.

Previous studies have investigated the combinatory effects of SFN and PTX in various cancer cell types, including breast [1], ovarian [2], lung [3], and prostate cancer [4]. These studies have consistently shown that the combination treatment can synergistically inhibit cell growth and induce apoptosis and that this effect is associated with an increase in ROS production and a decrease in Bcl2 expression. These findings suggest that SFN may have the potential as an adjuvant therapy to improve the efficacy of PTX in treating various types of cancer.

Prostate cancer is the sixth leading cause of death in men worldwide, with over one million men diagnosed with prostate cancer and over three hundred thousand deaths in 2012 alone. These numbers rank prostate cancer as the second most commonly diagnosed cancer in men [5]. The prostate gland secretes alkaline fluid in the male reproductive system as part of semen, which functions as a pH buffer to protect the sperm [6]. Enlargement of the prostate gland is a common age-related symptom known as benign prostatic hypertrophy (BPH), which, although noncancerous, can result in unpleasant clinical symptoms such as urination problems, infections, and kidney diseases [7]. The development of prostate cancer is gradual and occurs over a prolonged period, making early diagnosis challenging. The etiology of the disease is widely heterogeneous, with genetics, ageing, obesity, and ethnicity identified as major risk factors [8]. Recently, researchers have studied the role of dietary habits in the incidence of prostate cancer, highlighting high intakes of dairy products, red meats, processed meats, and foods rich in α-linolenic acid, and calcium as possible risk factors [9–11]. Such research has created a groundswell of interest in studying the influences of certain dietary components on human health and disease status.

Sulforaphane (SFN), a compound found naturally in cruciferous vegetables, has potential therapeutic properties, including detoxification, antimicrobial, anti-inflammatory, and redox balancing [12]. SFN’s therapeutic and protective properties may be attributed to the induction of the nuclear factor-erythroid-2-related factor 2 (Nrf2) transcription factor, which regulates antioxidant response elements, inflammation, non-enzyme antioxidants, and phase II detoxification enzymes [13]. SFN has been shown to induce the expression of quinone reductase and glutathione transferases, phase II anticarcinogenic enzymes, in murine hepatoma cells [14–16]. SFN has been focused on by researchers and utilized to treat different types of cancers due to its promising therapeutic properties [17].

The role of SFN in human prostate cancer was previously studied by researchers who reported potent induction of phase II enzymes and initiation of reactive oxygen species following SFN treatment [18, 19]. SFN has also shown a protective effect against prostate cancer recurrence and significantly lowered the prostate-specific antigen (PSA) progression after radical prostatectomy [20]. Additionally, SFN decreased upregulated histone deacetylase (HDAC3) protein expression in transgenic adenocarcinoma of the mouse prostate [21].

In cancer treatment, combined therapy results in synergistic or additive outcomes and reduces the development of drug resistance in response to anticancer agents compared with monotherapy [22]. Paclitaxel (Taxol, PTX**)** is an anticancer drug that targets actively dividing cells by halting their mitosis, arresting cell growth, and ultimately initiating apoptotic cell death [23]. Here, we compare the outcomes of combined and monotherapies of SFN and PTX in prostate cancer cell lines (PC-3 and LNCaP). We apply molecular biology techniques to measure apoptosis, cell cycle arrest, and the expression of Bax and Bcl2 proteins in response to the two different treatment strategies. Understanding the mechanism of drug synergy, as opposed to simply knowing which drugs to combine, enables further optimization of advantageous drug interactions and can provide efficient therapeutic strategies in preclinical research. Such research could lead to the development of new biomarkers and guide therapy choices, ultimately improving the treatment outcomes for patients with prostate cancer.

## Materials and methods

### Cell lines and reagents

The prostate cancer adenocarcinoma cell line PC-3 (catalog no. CRL-1435; ATCC) and prostate carcinoma cell line LNCaP (catalog no. CRL-1740; ATCC) were obtained from ATCC, Egypt. The cells were cultured in RPMI-1640 complete growth medium (catalog no. 12633-012; Gibco) supplemented with 10% heat-inactivated fetal bovine serum (FBS) (catalog no. 098105; Multicell). The seeded cells were incubated at 37 °C and 5% CO_2_ in a humidified incubator, and the medium was changed every 48 h. Once the cells reached 80% confluency, they were dissociated using 0.25% trypsin (catalog no. 15400-054; Gibco) and plated in 24-well cell culture plates at a density of 70,000 cells per well for 24 h before treatment.

Paclitaxel (PTX) (catalog no. T7402; Millipore Sigma) was dissolved in dimethyl sulfoxide (DMSO) (catalog no. d5879; Sigma-Aldrich) at a concentration of 50 mg/mL. Sulforaphane (SFN) (catalog no. s4441; Millipore-Sigma) was diluted in DMSO to a concentration of 5 mg/mL. Different concentrations of PTX or SFN were freshly prepared in a complete culture medium before treatment.

### Cell viability assay

To examine the effect of PTX and SFN on PC-3 cell viability, cells were grown in complete medium in 48-well plates at a density of 35,000 cells per well for 24 h before treatment. To investigate the effect of PTX in combination with SFN on PC-3 cell viability, we dissolved both drugs at equal concentrations starting at 100 ng/ml to 2500 µg/ml. Thiazolyl blue tetrazolium bromide (MTT) (catalog no. m-5655; Sigma) was used for the cell viability assay. MTT was dissolved in phosphate-buffered saline at a concentration of 5 mg/mL. After treating the cells with PTX, SFN, or PTX** + **SFN, MTT was added to each well of 24-well plates at a final concentration of 1 mg/mL directly to the culture medium. The plates were then incubated at 37 °C for three hours.

The culture medium was then removed, and the MTT formazan crystals were dissolved in 500 µL of MTT solvent (4 mM HCL, catalog no. acs393; BDH, 0.1% Nonidet P40, catalog no. 74,385; Fluka, in isopropyl alcohol, catalog no. un1219; Omnisolv) on a rocker in the dark for 15 min.

Then, 100 µL of the dissolved MTT crystals were transferred to each well in a 96-well plate and were read on a SPECTRAmax PLUS384 Microplate spectrophotometer set to a 590 nm wavelength. The absorbance values were used to calculate the percentage of viable cells relative to the untreated control cells.

### Cell lysate preparation, total protein quantification, and Western blot analysis

After the treatments, the culture medium was removed, and cells were harvested in Radioimmunoprecipitation assay buffer (RIPA) containing a 1x protease inhibitor cocktail (catalog no. PI-78439c; Thermo Scientific). Total protein quantification was conducted using the BioRad protein assay (catalog no. 500-0006; BioRad) following the manufacturer’s protocol.

Total protein was denatured by adding 2x Laemmli buffer (SDS, 4%; β-mercaptoethanol, 10%; glycerol, 20%; bromophenol blue, 0.004%; Tris-HCl, 0.125 M) in a 1:1 (v/v) ratio and boiled at 95 °C for 5 min. Then, 50 µg of protein per sample was loaded into 10% SDS**-**PAGE. BLUelf pre-stained protein ladder (catalog no. PM008-0500; Frogga Bio) was used as a molecular weight marker (5-245 kDa).

The separated protein bands were transferred to a nitrocellulose membrane (catalog no. rpn203D; EG Healthcare). The membranes were immune-probed with rabbit polyclonal anti-caspase-3 (catalog no. AAP-113E; Stressgen), rabbit monoclonal anti-Bax (catalog no. ab32503; Abcam), rabbit monoclonal anti-Bcl2 (catalog no. ab32124; Abcam), and anti-glyceraldehyde-3-phosphate dehydrogenase (anti-GAPDH) (catalog no. 4699–9555; Biogenesis).

To detect the immune-probed protein bands, we used peroxidase-affiniPure goat anti-mouse IgG (catalog no. 115-035-003; Jackson ImmunoResearch) or peroxidase-conjugated goat anti-rabbit IgG (catalog no. 111-035-003) as secondary antibodies. Band visualization and densitometric analysis were carried out using Pierce ECL Western Blotting Substrate (catalog no. PI-32,106; Thermo Fisher Scientific), Chemi Doc XRS system, and Image Lab 6.0 software (BioRad).

### Fluorescent microscopy and image analysis

Cells were grown on coverslips placed at the bottom of each well in 24-well plates at a density of 70,000 cells per well for 24 h before treatment.

Following treatment, the cells were fixed in ice-cold methanol (catalog no. a412; Fisher Chemicals) for 10 min at -20 °C. Methanol treatment permeabilized the cell membrane and allowed 4’,6-diamidino-2-phenylindole (DAPI, catalog no. d21490; Molecular Probes) to penetrate and stain the nuclear chromatin. DAPI was prepared in phosphate-buffered saline (PBS) at a concentration of 300 nM and added to the cell monolayers for 5 min, followed by three washes (5 min each) in PBS.

Cells were then mounted using prolonged gold anti-fade reagent (catalog no. p36930; Invitrogen) and visualized with confocal microscopy (Zeiss, Oberkochen, Germany) using ZEN 2012 software. Images were acquired using appropriate filter settings and were analyzed using ImageJ software for the quantification of nuclear DAPI staining. The number of fluorescently stained nuclei was counted per field of view, and the average number of stained nuclei was calculated.

### Propidium iodide (PI) staining and cell cycle analysis

After treatment, PC-3 cells were fixed for 30 min in 70% ethanol at 4 °C. The cells were then washed twice in PBS, and 100 µg/ml of RNase A (catalog no. 1,007,885; Qiagen) was added, followed by incubation for 20 min at 37 °C. The cells were then washed twice with PBS. Next, the cells were incubated in 3 µM PI (catalog no. P4170; Sigma) in staining buffer (100 mM Tris, pH 7.4, 150 mM NaCl, 1 mM CaCl2, 0.5 mM MgCl2, 0.1% Nonidet P40) for 15 min at room temperature. Cell cycle analysis was carried out using a flow cytometer (Guava® easyCyte; Millipore Sigma). Data acquisition and analysis were carried out using Guava cell cycle data acquisition and analysis software (Guava Technologies).

### Annexin V and PI dual staining and flow cytometry analysis

PC-3 cells were harvested and washed twice with ice-cold phosphate-buffered saline (PBS). The cells were then resuspended in annexin V binding buffer (10 mM HEPES, pH 7.4, 140 mM NaCl, 2.5 mM CaCl2) at a concentration of 1 × 10^6 cells/ml. Annexin V-FITC conjugated (catalog no. A13199; Thermo Fisher) was added at a dilution of 1:100, and the cells were incubated in the dark for 15 min at room temperature.

Following Annexin V staining, the cells were washed twice with the Annexin V binding buffer and resuspended in the same buffer. PI (final concentration of 3 µM) was added, and the cells were incubated in the dark for 15 min at room temperature. After PI staining, the cells were washed twice with the annexin V binding buffer and fixed in 1% formaldehyde prepared in the Annexin V binding buffer for 10 min on ice. The fixed cells were then washed twice with PBS, and RNase A was added at a final concentration of 100 µg/ml. The cells were incubated for 20 min at 37 °C to digest RNA.

Prior to flow cytometry analysis, the cells were washed twice with PBS and resuspended in 500 µL of PBS. Annexin V/PI positivity and data analysis was conducted using a flow cytometer (Guava® easyCyte; MilliporeSigma) and Guava data acquisition and analysis software (Guava Technologies).

Cell populations were classified into four categories based on the staining pattern: viable cells (Annexin V-/PI-), early apoptotic cells (Annexin V+/PI-), late apoptotic or necrotic cells (Annexin V+/PI+), and necrotic cells (Annexin V-/PI+). The percentage of cells in each population was determined by gating on the appropriate regions of the Annexin V/PI dot plot.

### Statistical analysis

Data were obtained from n independent biological experiments and are presented as the mean of individual values with standard deviation (**SD**) error bars or as box-and-whisker plots showing the median, the 25th and 75th quartiles, as well as the minimum and maximum values.

To evaluate synergistic effects, the methodology established by Slinker et al. [24] was employed. Synergy was determined based on the criterion that the combined treatment effect (PTX + SFN) should surpass the cumulative effect of the individual drugs, as indicated by the equation:

$$\left[\mathrm{Effect}\;\left(\mathrm{PTX}+\mathrm{SFN}\right)\;>\;\mathrm{Effect}\;\left(\mathrm{PTX}\right)\;+\;\mathrm{Effect}\;\left(\mathrm{SFN}\right)\right]$$

Western blot densitometric analysis was carried out using the ChemiDoc XRS system, Image Lab 6.0 (Bio-Rad), and TotalLab TL120. GraphPad Prism 6 and Microsoft Excel software were used for statistical analyses and graph generation.

Student’s t-test was used to compare two groups, and one-way analysis of variance (ANOVA) with Tukey’s correction was used for multiple comparisons to determine the significant difference between PTX, SFN, PTX **+ **SFN, and the PC-3 non-stimulated cells (NS) that had not received any therapies.

## Results

### Cell viability, dose-response analysis, and SFN effect on antiproliferation of PTX in PC-3 cells

Different concentrations of PTX or SFN were prepared as indicated in the Methods section, and the cells were treated with the drugs.

**Fig. 1:**
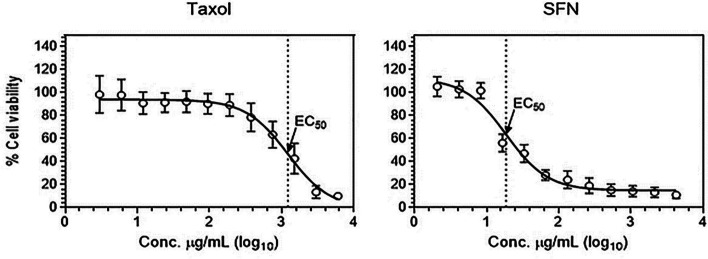
shows the dose-response curves of PTX and SFN on PC**-**3 cell viability. The EC_50_ values for PTX and SFN were calculated, and the data are presented as mean values with ± SD error bars. The MTT assay was used to measure cellular metabolic activity, and the data were obtained from four independent experiments

MTT assay was conducted 24 h after treatment, and the percentage of cell viability was determined for each drug concentration. Our data revealed that both PTX and SFN significantly reduced PC-3 cell viability in a dose-dependent manner (Fig. 1). TheEC_50_ value for PTX was higher (1.2 mg/ml) than that for SFN-treated cells (18.7 µg/ml).

### The SFN synergized PTX effect on reducing PC-3 cell viability

**Fig. 2:**
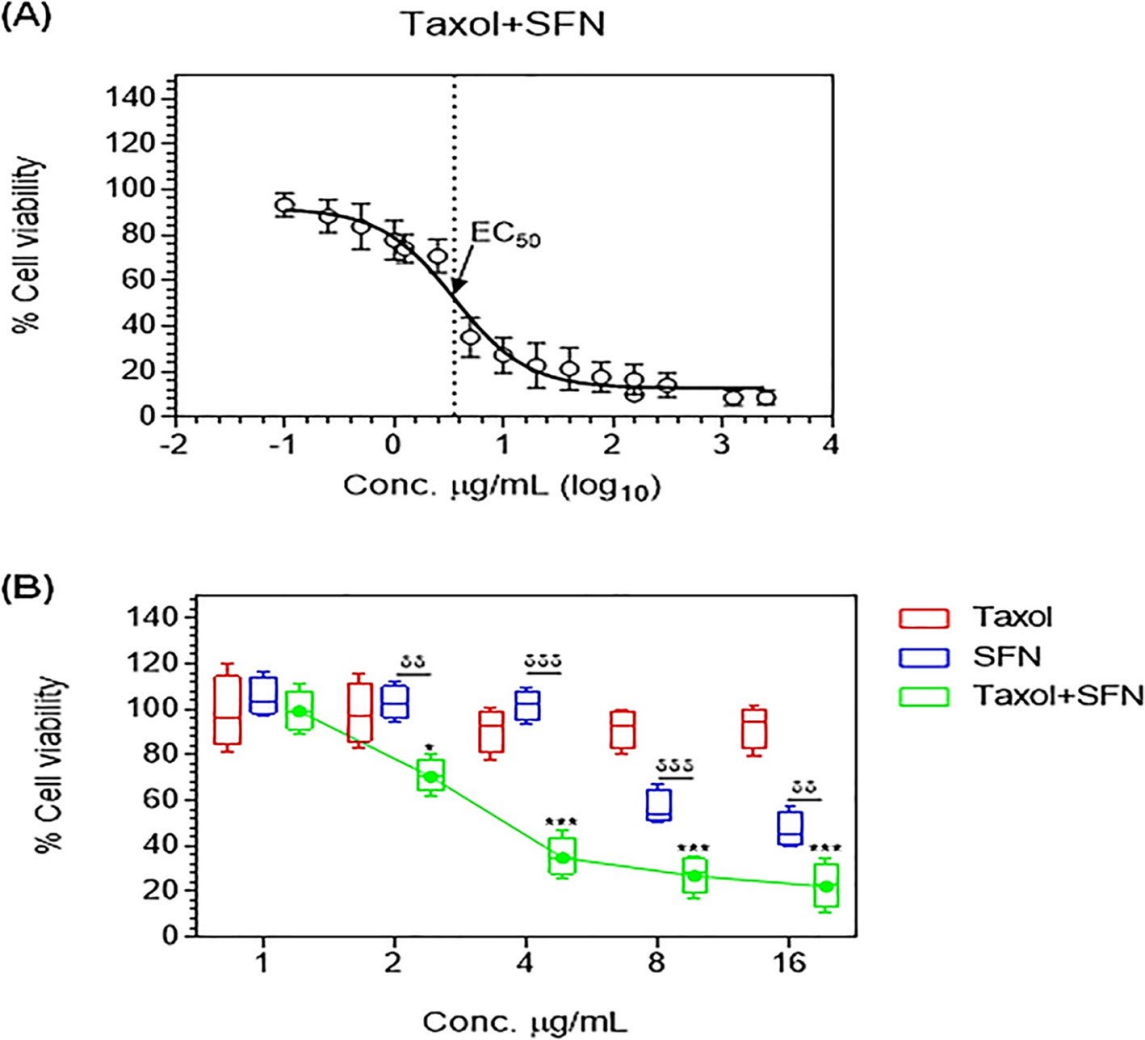
shows the synergistic effect of SFN and PTX on reducing cell viability in PC**-**3 cells. The EC_50_ value of the combined treatment was determined by treating cells with equal concentrations of  PTX and SFN and conducting the MTT cell viability assay 24 h after treatment. The data were obtained from five independent experiments and are presented as mean values with ± SD error bars in (**A**) or box-and-whisker plots in (**B**). Student’s t-test was used to determine the significant difference between PTX and PTX** + **SFN or between SFN and PTX** + **SFN. The data show that the combination treatment of SFN and PTX had a synergistic effect on reducing cell viability in PC**-**3 cells. (*p ≤ 0.05, **p ≤ 0.01, ***p ≤ 0.001)

Our results showed that the combination treatment synergized the effects of both drugs on reducing cell viability. The EC_50_ value for the combination was 3.5 µg/ml, which was 342-fold and 5.3-fold lower than the EC_50_ values for PTX and SFN individual treatments, respectively (Fig. 2A).

In a separate experiment, we compared the percentages of viable cells following treatment with PTX or SFN individually and in combination. Our results demonstrated that the PTX and SFN combination had a significantly more potent effect on reducing PC**-**3 cell viability, even at a low concentration of 2 µg/ml. At this concentration, the PTX and SFN combination significantly reduced cell viability to 70.86% (*p* ≤ 0.013) compared to the PC**-**3 non-stimulated cells, which were considered 100%. In contrast, at the same concentration of 2 µg/ml, neither PTX nor SFN individual treatments showed significant effects on reducing the percentage of viable PC**-**3 cells (*p* ≥ 0.05) (Fig. 2B). The effect of the combination treatment continued to increase with increasing drug concentrations up to 8 µg/ml, and higher concentrations did not result in further improvements in reducing cell viability.

### A combination of PTX and SF Ninduces Caspase-3activation and nuclear morphology changes characterizing apoptosis in PC-3cells

Caspase 3 activation and changes in cell nuclei morphology, such as nuclear fragmentation and micronuclei appearance, are hallmarks of apoptotic cell death. To evaluate the effect of PTX and/or SFN treatments on inducing apoptosis in PC-3 cells, we treated the cells as described in the Methods section. We utilized western blot analysis and fluorescent microscopy to detect caspase 3 activation and nuclear morphological changes.

**Fig. 3:**
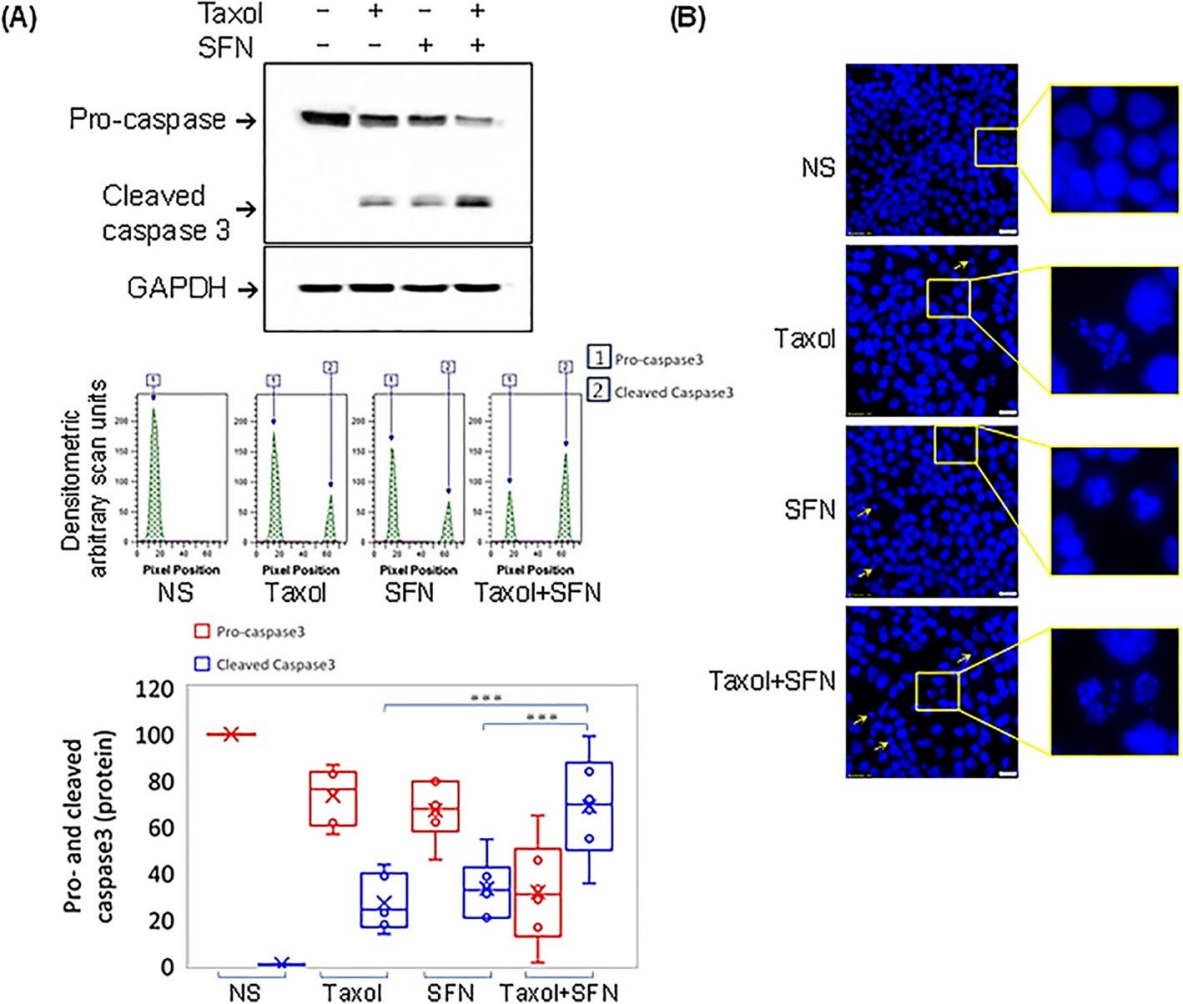
shows that SFN additively enhances PTX-induced apoptosis in PC**-**3 cells. **A** Western blot and densitometric analysis of caspase-3 protein bands revealed significantly higher band intensities of cleaved caspase**-**3 in PTX **+ **SFN-treated cells compared to PTX or SFN alone. This was accompanied by a reduction in pro**-**caspase**-**3 bands. **B** Fluorescent micrographs of DAPI-stained nuclei showed nuclear fragmentation and micronuclei formation in cells treated with PTX, SFN, or PTX **+ **SFN. The data were obtained from five independent experiments and are presented as box-and-whisker plots for pro**-**caspase**-**3 and activecaspase**-**3 band intensities. Student’s t-test was used to determine the significant difference between PTX or SFN individual treatments and PTX** + **SFN combined treatments. The data suggest that SFN enhances the apoptotic effect of  PTX in PC**-**3 cells. (***p ≤ 0.001)

As expected, treatment with PTX or SFN resulted in the cleavage of pro-caspase 3 protein into smaller active caspase 3 subunits, which was detected by western blot at ~ 17 kDa. Densitometric analysis of caspase 3 protein bands revealed that the intensity of cleaved caspase 3 bands in the protein lysate of PC-3 cells treated with the PTX and SFN combination was significantly higher than in the protein lysates of cells treated with PTX or SFN individually (Fig. 3A).

In addition to caspase 3 activation, we observed significant nuclear morphology changes in PC**-**3 cells treated with the PTX and SFN combination. Specifically, we observed nuclear fragmentation and micronuclei appearance, which are characteristic of apoptotic cell death. These changes were detected using fluorescence microscopy and were not observed in cells treated with PTX or SFN alone.

Microscopic visualization of DAPI-stained nuclei showed morphological changes, including chromatin condensation, micronuclei, and nuclear fragmentation, along with a noticeable reduction in the number of nuclei in the visualized fields, likely due to cell detachment after treatments. These changes were not observed in non-stimulated PC**-**3 cells, which retained a normal round nuclei appearance (Fig. 3B). Furthermore, the apoptotic cell death characteristics were more pronounced in cells treated with the PTX** + **SFN combination compared to either agent alone. These findings suggest that SFN enhances the apoptotic effect of PTX in PC**-**3 cells.

### The combination effect of PTX and SFN on redistributing the cell-cycle growth phases in PC-3

**Fig. 4:**
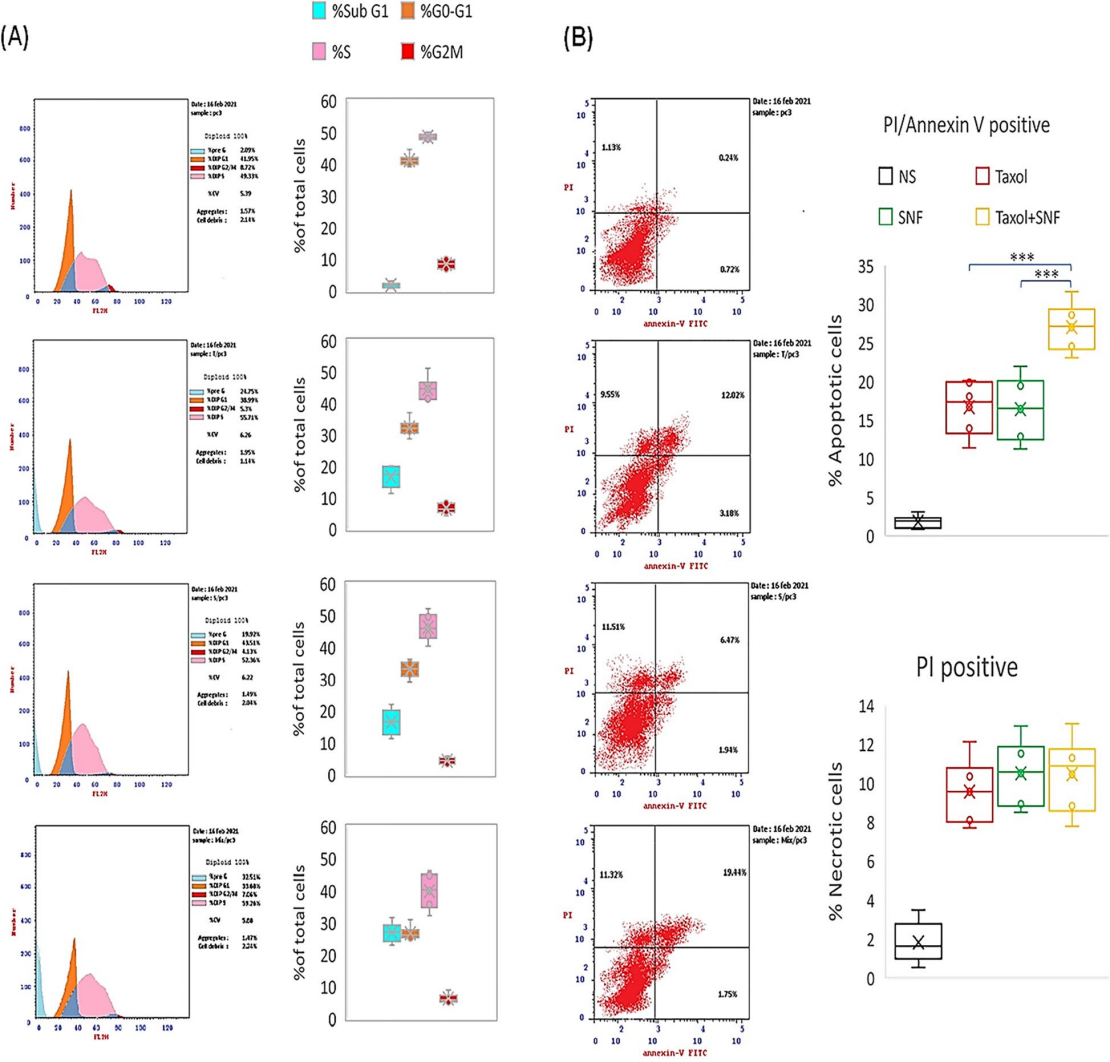
shows that SFN enhances PTX-induced cell-cycle arrest and apoptosis without affecting the number of necrotic PC**-**3 cells. The flow cytometry analysis of cell-cycle growth phases demonstrated that PTX or SFN treatments increased the sub-G1 population, indicating apoptotic cell death. The combination treatment of PTX and SFN showed a greater effect than either agent alone. The scatter plots of cells double-stained with propidium iodide and Annexin V showed that both PTX and SFN treatments increased the number of necrotic cells compared to non-stimulated cells. However, the combination treatment did not show a significant increase in the number of necrotic cells compared to the individual treatments. These results suggest that SFN enhances the apoptotic effect of PTX in PC**-**3 cells without increasing the number of necrotic cells. The data were obtained from five independent experiments and are presented as box-and-whisker plots for cell-cycle analysis and scatter plots for apoptotic (**A**) and necrotic (**B**) effects. Significant differences were determined using the Student’s t-test for two-group comparisons. (***p ≤ 0.001)

To investigate the effect of PTX and/or SFN on the cell cycle growth phases in PC**-**3 cells, we stained the cells with propidium iodide and analyzed them using flow cytometry according to the methods section. Treatment with PTX or SFN increased the percentage of the sub-G1 population by 9.23-fold (*p* ≤ 0.0002) or 9.10-fold (*p* ≤ 0.0006), respectively, compared to non-stimulated cells. The effect of the combined treatment was statistically more significant than PTX or SFN alone and increased the sub-G1 population by 14.98-fold (*p* ≤ 0.0001) compared to non-stimulated cells. This increase was 1.6-fold (*p* ≤ 0.003) and 1.7-fold (*p* ≤ 0.002) higher than PTX or SFN alone, respectively (Fig. 4A). An increasing sub-G1 population is indicative of apoptotic cell death, and our data confirmed that this effect was augmented when cells received the PTX** + **SFN combined treatment.

Furthermore, PTX or SFN induced an S-phase growth arrest by 6.38% (*p* ≤ 0.01) or 3.1% (*p* ≤ 0.05), respectively. The combination treatment enhanced this effect to reach 9.93% (*p* ≤ 0.002).

To evaluate the necrotic effects of PTX and/or SFN, we double-stained PC**-**3 cells with propidium iodide and Annexin V and counted the necrotic cells using flow cytometry. Our results showed that PTX or SFN treatments increased the number of necrotic cells by 5.23-fold (*p* ≤ 0.0002) or 5.74-fold (*p* ≤ 0.0003), respectively, compared to non-stimulated cells. Interestingly, there was no significant difference among the fold-change values of necrotic cells in PTX, SFN, and the combination treatments (*p* ≥ 0.05). The combined treatment increased the number of necrotic cells to 5.71-fold (*p* ≤ 0.0002) compared to the number of necrotic cells in non-stimulated PC**-**3 cells, which is similar to the fold-change values of  PC**-**3 cells treated individually with PTX or SFN (Fig. 4B).

### The combination of PTX and SFN had a stronger effect on modulating Bax and Bcl_2_ protein expression

Protein lysates were prepared from the PC**-**3 cell line and separated using SDS**-**PAGE. The protein bands were then transblotted to nitrocellulose membranes and probed with antibodies against Bax, Bcl2, and GAPDH. The visualization and densitometric analysis of the bands showed significant increases in Bax protein expression by 185.08% (*p* ≤ 0.04) or 224.56% (*p* ≤ 0.01) following PTX or SFN treatments, respectively. An additive effect was observed on increasing Bax protein levels when cells were subjected to the combined treatment by 353.56% (*p* ≤ 0.0002). Bcl2 showed an opposite expression pattern to Bax, where PTX or SFN reduced Bcl2 expression in PC**-**3 cells. An additive effect was also observed in reducing Bcl2 levels after the combined treatments.

**Fig. 5:**
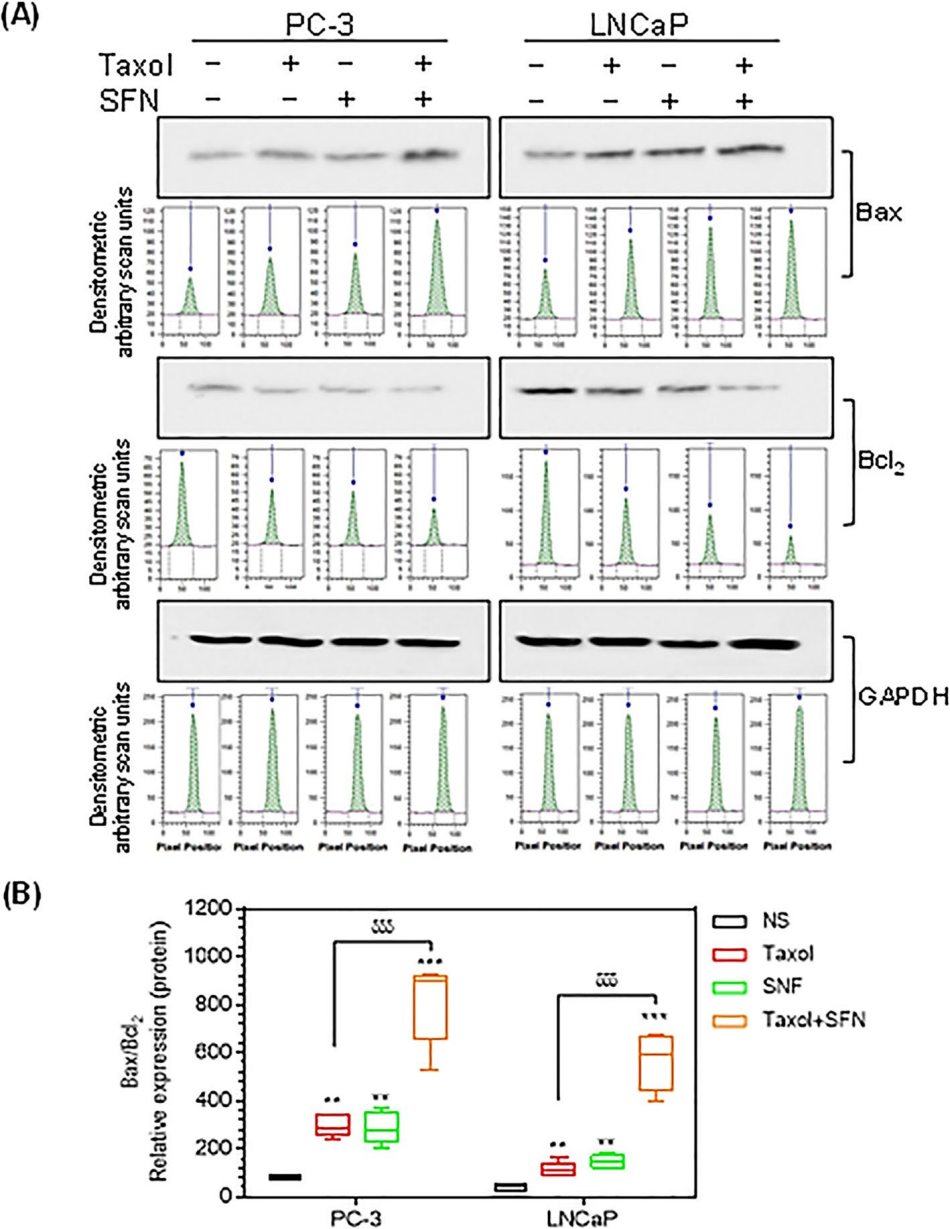
shows the effect of PTX, SFN, or PTX **+ **SFN treatments on the expression of Bax and Bcl2 proteins in PC**-**3 and LNCaP cells. **A** The Western blot and densitometric analysis histograms demonstrate significant differences between non-stimulated cells (NS) and PTX, SFN, or PTX** + **SFN-treated cells. **B** The Bax:Bcl2 ratios in PC**-**3 and LNCaP cells are significantly increased with PTX, SFN, or PTX** + **SFN treatments. The statistical analysis presented demonstrates the significance of the differences observed. The data provide further support for the additive effects of PTX and SFN on inducing apoptosis in prostate cancer cells. (**p ≤ 0.01, ***p ≤ 0.001)

To further investigate, we calculated the ratio between Bax and Bcl2 protein expression and found a significant increase in such ratios in PC**-**3 cells treated with PTX or SFN compared to non-stimulated cells by 3.54-fold (*p* ≤ 0.0007) or 3.4-fold (*p* ≤ 0.002), respectively. The PTX** + **SFN combined treatment increased the Bax**/**Bcl2 ratio to 9.68-fold (*p* ≤ 0.0006) (Fig. 5A & B; Table **S1**).

To confirm our findings, we treated another prostate cancer cell line, LNCaP, with PTX, SFN, or the combination of PTX** + **SFN, as described in the methods section. The data collected from LNCaP cells confirmed that PTX or SFN increased the protein expression of Bax and reduced Bcl2 levels, thus increasing the ratio of Bax**/**Bcl2. These effects were augmented with the combined treatment of  PTX** + **SFN (Fig. 5A & B; Table  S2).

## Discussion

Although boswellic acid, sulforaphane, and ginsenoside have demonstrated remarkable anticancer activity and are being considered potential clinical candidates [25], it is worth noting that there are currently around 10,000 active clinical trials in the United States investigating combination therapies for a range of conditions, including cancer, infectious diseases, metabolic disorders, cardiovascular diseases, autoimmune disorders, and neurological disorders.

Combination therapies are compared to single drugs and are considered effective if they produce a better response when administered together than if they are taken individually. However, because many drug combinations could have additive or even synergistic effects, the degree of synergy becomes the most important factor to consider. To answer this, the two-drug combination should be compared to not only single treatments but also the best of previously reported drug combinations [26].

PTX has been used as an anticancer agent for treating a variety of cancers since 1989; its therapeutic outcomes did not meet the high expectations of physicians, rather than patients; because many types of cancers, such as prostate, breast, and ovarian cancers resist PTX treatment [27, 28]. In our endeavors to improve the anticancer potency of  PTX and SFN, another promising and naturally existing phytochemical found primarily in green cruciferous vegetables, may result in better treatment outcomes in prostate cancer cell models. Since 1987, when Levin and Hryniuk [29] introduced the concept of drug dose intensity, the dose, and duration of a given drug are precisely calculated to deliver the maximum tolerated dose to overcome resistance to such chemotherapeutic agents. High drug doses are most likely accompanied by severe side effects, which is why researchers aspire to any therapeutic regimens that would lead to lowering the dose and consequently the side effects and maintaining the efficacy of the drug. The data presented here showed that combining PTX and SFN dramatically lowered the EC_50_ of both drugs compared to the EC_50_ values of the same drugs when administrated individually. If applied to prostate cancer patients, such findings may benefit them and help avoid or attenuate the side effects of high doses.

Apoptosis is a natural process through which the body eliminates unwanted and damaged cells. A balanced apoptosis/cell division rate protects from the occurrence of uncontrolled cell division and ultimately the development of cancers [30]. Unlike necrosis, apoptotic cell death does not elicit an inflammatory response and allows the body to recycle the dead cell contents efficiently [31]. In cancer, chemotherapeutic drugs retrieve the balance between apoptotic and survival signals by specifically targeting cells with a high division rate. PTX is known to halt mitotic division leading to cell cycle arrest and apoptosis [32]; in our current study, we evaluated the effect of SFN on PTX-induced cell cycle arrest and apoptosis in the PC**-**3 prostate cancer cell line. Our data confirmed that SFN significantly improved PTX-induced activation of caspase-3 and increased the number of accumulated cells in the S-growth phase (S-growth phase arrest). Such changes were accompanied by the appearance of apoptotic bodies and micro-nuclei in the treated cells. Interestingly, PTX** + **SFN combined treatment did not increase the number of necrotic cells comparable to PTX or SFN individual treatments. These data indicate that SFN augmented the potency of PTX as an anticancer agent and at the same time did not increase necrosis, which is one of PTX’s side effects.

One of the strategies that a cancer cell adopts to evade apoptosis is to upregulate the expression of survival signaling proteins, such as Bcl_2_, which showed higher levels in prostate cancer [33, 34]. The elevated Bcl_2_ mRNA and protein levels were specifically reported in the prostate cancer cell line (LNCaP) [34]. Higher levels of Bcl_2_ inhibit caspase activities and result in resistance to apoptosis by preventing the release of cytochrome *c* from the mitochondria [35]. Additionally, other researchers reported that Bcl_2_ binds to the apoptosis-activating factor (APAF**-**1) [36]; therefore, we proposed that lowering Bcl_2_ levels may sensitize transformed cells to anticancer drugs, such as PTX. The data presented here confirmed that PTX in combination with SFN additively reduced the level of Bcl_2_ protein compared with PTX or SFN alone in both prostate cancer cell models (PC**-**3 and LNCaP). This result is consistent with previously published reports, where inhibiting Bcl2 using nonpeptide small molecule inhibitors improved the therapeutic outcomes for targeting prostate cancer cells [37, 38]. Furthermore, the ratio of Bax (pro-apoptotic Bcl_2_ family member): Bcl_2_ protein levels are crucial for cell survival [39], where increasing Bax levels overcome the threshold that Bcl_2_ can neutralize, Bax, translocation to the mitochondria, leading to the release of cytochrome *c* and trigger apoptosis [35]. So we investigated the levels of Bax protein following different treatments and calculated the Bax:Bcl_2_ ratio. Our data indicated that combination therapy of PTX and SFN significantly increased Bax protein expression and Bax:Bcl_2_ ratio compared to PTX or SFN individual treatments in both PC**-**3 and LNCaP cell lines.

### Limitations of the combination treatment of PTX and SFN compared to individual treatments in both PC-3 and LNCaP cell lines

One limitation is the potential for increased toxicity when two drugs are combined. While the combination treatment of PTX and SFN was found to lower the EC_50_ of both drugs compared to individual treatments, the combination treatment could lead to increased toxicity in vivo. Therefore, careful dose optimization and toxicity studies are needed to ensure that the combination treatment is safe and effective.

Another limitation is the potential for drug resistance to develop with long-term use of the combination treatment. While the combination treatment of PTX and SFN has been shown to improve therapeutic outcomes in prostate cancer cell models, cancer cells could develop resistance to the combination treatment over time. Therefore, in clinical trials, it will be important to monitor patients receiving this combination treatment for signs of drug resistance and to develop strategies to overcome resistance if it develops.

Finally, it is important to note that the results of this study were obtained in vitro, and the efficacy of the combination treatment of PTX and SFN in vivo may differ from the results seen in cell culture. Therefore, further preclinical studies and clinical trials will be needed to determine the safety and efficacy of this combination treatment in vivo.

### The synergistic mechanisms through which SFN enhances the efficiency of PTX

1. Highly effective anti-cancer therapy: Our findings demonstrate that the combination of  PTX and SFN reduces resistance to PTX and allows for the use of lower doses of both PTX and SFN metabolites for effective anti-cancer treatment as reported by Wang [40].
2. Enhanced Efficacy: Combining PTX with SFN has demonstrated improved treatment outcomes and an increased likelihood of tumor regression. The synergistic effect observed in the study resulted in a higher level of apoptosis compared to individual treatments. The combination therapy induced characteristic features of apoptotic cell death, such as nuclear fragmentation and the appearance of micronuclei. These morphological changes indicate that the combination treatment effectively promotes cancer cell death [41].
3. Caspase-3 Activation: Caspase-3 plays a crucial role in the execution phase of apoptosis. Our results indicated that the combination treatment of PTX and SFN induces the activation of caspase-3. Caspase-3 activation leads to the cleavage of various target proteins, resulting in the dismantling of cellular structures, causing microtubule degradation, and ultimately leading to cell death, as evident in both clinical and preclinical trials [3, 40, 41].
4. Cell Cycle Modulation: Treatment with PTX or SFN alone increased the sub**-**G1 population, representing cells undergoing apoptotic cell death in human bladder cancer T24 cells [42]. Our data demonstrate that the combination treatment further enhances this effect, indicating a higher rate of cancer cell death. Moreover, both PTX and SFN individually induce S**-**phase growth arrest, and our current work confirms that the combination treatment amplifies this effect. S**-**phase arrest prevents cancer cells from replicating their DNA and progressing through the cell cycle, ultimately leading to cell death [43].
5. Modulation of Bax and Bcl2 Protein Expression: Our study reveals that PTX and SFN individually increase the expression of Bax and decrease the expression of Bcl2. This modulation of Bax and Bcl2 protein expression promotes the pro-apoptotic signaling cascade, favoring apoptosis. An elevated Bax**/**Bcl2 ratio signifies a shift towards pro-apoptotic signaling, leading to improved therapeutic outcomes [44, 45].

Because SFN-induced transcriptomic changes depend on the cell/tissue type, little is known about the context-dependent effects of SFN [46]. Thus, it is important to note that the precise mechanisms by which SFN enhances PTX efficiency may vary depending on the specific cancer type and cellular context. Therefore, further research is necessary to fully comprehend the mechanisms underlying the synergistic effects of SFN and PTX in different types of cancer.

### Are there any clinical trials investigating the efficacy of SFN and PTX combination therapy?

There are currently no completed clinical trials investigating the efficacy of SFN and PTX combination therapy in cancer patients. However, some ongoing clinical trials are investigating the use of SFN and PTX in combination with other treatments. Here are some examples:

1. A phase II clinical trial is currently investigating the efficacy of a combination of PTX, SFN, and cisplatin in patients with advanced non-small cell lung cancer (NSCLC) who have not received prior chemotherapy. The study aims to evaluate the safety and efficacy of the combination treatment, and the primary endpoint is the overall response rate [47].
2. Another phase II clinical trial is investigating the efficacy of a combination of PTX, SFN, and carboplatin in patients with advanced ovarian cancer who have received prior platinum-based chemotherapy. The study aims to evaluate the safety and efficacy of the combination treatment, and the primary endpoint is progression-free survival [48].
3. A phase I clinical trial is investigating the safety and tolerability of a combination of SFN and PTX in patients with advanced solid tumors. The study aims to determine the maximum tolerated dose of the combination treatment, and the primary endpoint is dose-limiting toxicity [49].

It should be noted that these clinical trials are currently ongoing and their results are not yet available. Therefore, it is unclear whether SFN and PTX combination therapy will be effective in treating cancer patients in a clinical setting. However, these trials do suggest that SFN and PTX combination therapy is a promising approach for cancer treatment, and further clinical studies are needed to fully evaluate its safety and efficacy.

### The potential side effects of SFN and PTX combination therapy

They may include those associated with each drug individually, as well as potential synergistic effects.

1. Nausea and vomiting: Both PTX and SFN can cause nausea and vomiting. PTX is known to be a highly emetogenic drug, and SFN has been reported to cause nausea in some individuals [2, 50].
2. Neuropathy: PTX can cause peripheral neuropathy, which is characterized by numbness, tingling, and pain in the hands and feet. SFN has been shown to have neuroprotective effects, but it is unclear whether it can prevent or mitigate PTX-induced neuropathy [51, 52].
3. Myelosuppression: PTX can cause myelosuppression, which is a decrease in the number of blood cells produced by the bone marrow. SFN has been shown to have hematopoietic effects, but it is unclear whether it can prevent or mitigate PTX-induced myelosuppression [53, 54].
4. Liver toxicity: SFN has been shown to have hepatoprotective effects, but the combination treatment of SFN and PTX could increase the risk of liver toxicity [55, 56].
5. Drug interactions: SFN has been shown to induce phase II detoxification enzymes, which can increase the metabolism of drugs metabolized by these enzymes [57]. This could potentially decrease the efficacy of other drugs taken concurrently with PTX and SFN, or increase the toxicity of these drugs.

## Conclusion

The combination therapy of PTX and SFN holds great promise for improving treatment outcomes in prostate cancer. This study demonstrated that the combination of PTX and SFN resulted in a synergistic effect, significantly lowering the effective concentrations of both drugs compared to individual treatments. This combination therapy has the potential to benefit prostate cancer patients by reducing side effects while maintaining drug efficacy.

The mechanism of action underlying the improved therapeutic outcomes of the PTX and SFN combination therapy was investigated. The data showed that SFN improves the efficacy of PTX by enhancing apoptosis, activating caspase**-**3, inducing nuclear morphology changes, modulating the cell cycle, and altering the expression of Bax and Bcl2 proteins. These findings provide mechanistic insights into the synergistic effects of PTX and SFN, supporting the potential optimization of combination therapy for prostate cancer treatment.

The preclinical evidence and mechanistic insights gained from the PTX and SFN combination therapy study provide a foundation for further research and clinical investigations. Future studies could explore the combination therapy’s efficacy in different cancer types, evaluate its potential in combination with other treatments, and investigate the underlying mechanisms in various cellular contexts.

However, there are several limitations to consider. Firstly, the potential for increased toxicity associated with the combination treatment requires careful dose optimization and toxicity studies to ensure safety and effectiveness. Secondly, the development of drug resistance with long-term use of combination therapy is a concern, highlighting the need for ongoing monitoring and strategies to overcome resistance. Lastly, the results obtained *in vitr*o may not fully reflect the efficacy of the combination treatment in vivo, necessitating further preclinical and clinical trials.

### Supplementary Information

**Supplementary Material 1.**

## Data Availability

The datasets used and/or analyzed during the current study are available from the corresponding author upon reasonable request.

## Abbreviations

SFN: sulforaphane
PTX: Paclitaxel/ or Taxol
SFN: Sulforaphane
BPH: Benign prostatic hypertrophy
Nrf2: Nuclear factor-erythroid-2-related factor 2
PPAR-γ: Peroxisome proliferator-activatedγ
PSA: Prostate-specific antigen
HDAC3: Upregulated histone deacetylase protein
DMSO: Dimethyl sulfoxide
MTT: Thiazolyl blue tetrazolium bromide
RIPA: Radioimmunoprecipitation assay buffer
SDS-PAGE: Sodium dodecyl sulfate-Polyacrylamide gel electrophoresis
anti-GAPDH: anti-glyceraldehyde-3-phosphate dehydrogenase
DAPI: 4’,6-diamidino-2-phenylindole
PBS: Phosphate-buffered saline
PI: Propidium Iodide
APAF: Apoptosis-activating factor
