# Supplementary material for: Optimizing combination therapy in prostate cancer: mechanistic insights into the synergistic effects of Paclitaxel and Sulforaphane-induced apoptosis
Source: BMC Mol Cell Biol. 2024 Mar 4;25:5. doi: 10.1186/s12860-024-00501-z (PMC10910811; doi:10.1186/s12860-024-00501-z)
Supplement: Supplementary file 1 — Supplementary Material 1. [file 12860_2024_501_MOESM1_ESM.zip › Figure S3A.pdf]

## Figure S5 (A)

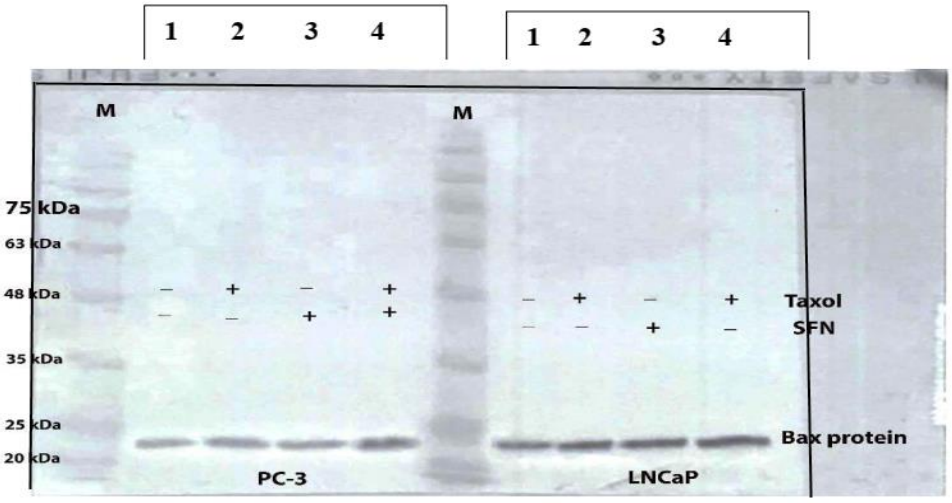


Expression of **Bax** prot[illegible]
(2) Cells treated with **PTX**, (3) Cells treated with **SFN**, and (4) Cells treated with **PTX+SFN**.

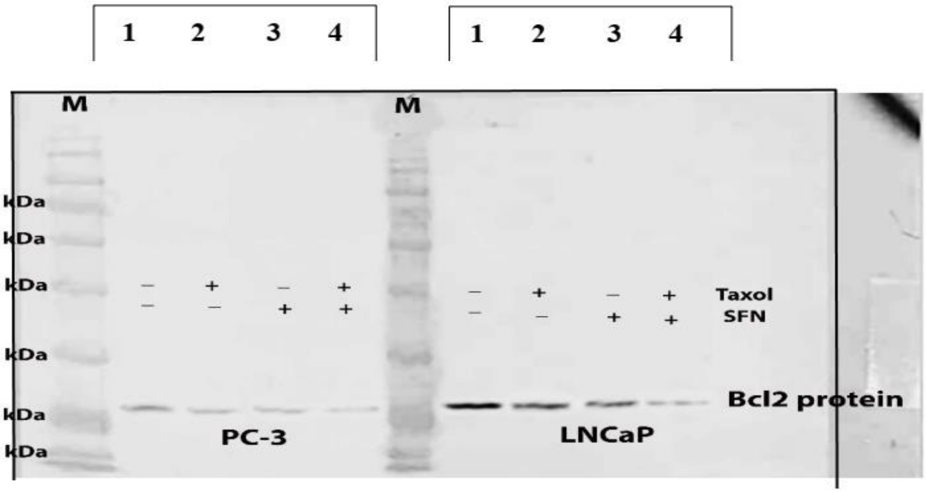


Expression of **Bcl2** protein in **PC-3** and **LNCaP** cells under different cellular conditions: (1) Non-stimulated cells (**NS**), (2) Cells treated with **PTX**, (3) Cells treated with **SFN**, and (4) Cells treated with **PTX+SFN**.

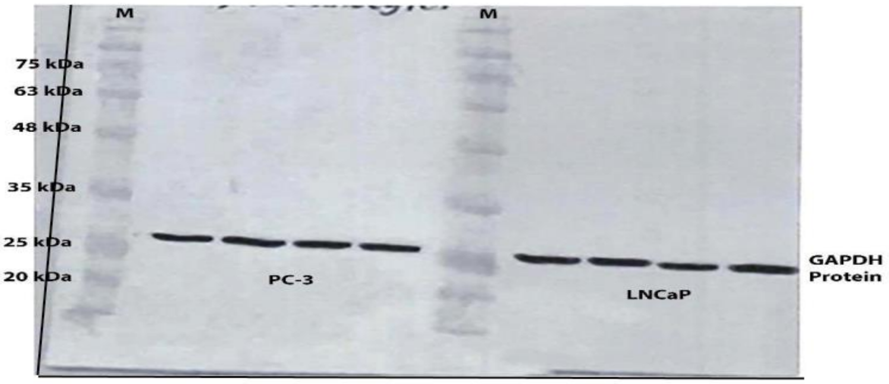


**GAPDH** was used as a loading control.
