# Supplementary material for: Optimizing combination therapy in prostate cancer: mechanistic insights into the synergistic effects of Paclitaxel and Sulforaphane-induced apoptosis
Source: BMC Mol Cell Biol. 2024 Mar 4;25:5. doi: 10.1186/s12860-024-00501-z (PMC10910811; doi:10.1186/s12860-024-00501-z)
Supplement: Supplementary file 1 — Supplementary Material 1. [file 12860_2024_501_MOESM1_ESM.zip › Table S1.pdf]

Table S1. presents the densitometric arbitrary scan units of the protein bands normalized to **GAPDH** for **Bax** and **Bcl2** protein expression in response to **PTX**, **SFN**, or **PTX+SFN** in the **PC-3** prostate cancer cell line. The statistical analysis performed shows significant increases in **Bax** protein expression and decreases in **Bcl2** protein expression in treated cells compared to non-stimulated control cells (**NS**) and among the mono- and combined treatments. The data suggest that **PTX** and **SFN** have additive effects on inducing apoptosis in **PC-3** cells, and the combination treatment may have a synergistic effect.

**Treatment**	**Proteins**	**Replicates**					**Mean**	**% Change relative to control (NS)**
		**1**	**2**	**3**	**4**	**5**		
**Control (NS)**	**Bax**	18.219	15.741	12.053	22.415	8.834	**15.452**	--
	**Bcl_2**	20.183	19.178	17.072	26.614	9.700	**18.549**	--
**PTX**	**Bax**	27.247	36.896	37.402	22.455	19.00	**28.600**	185.5, ($p \leq 0.04$)*
	**Bcl_2**	10.150	10.852	10.839	7.856	7.820	**9.503**	-48.7, ($p \leq 0.03$)*
**SFN**	**Bax**	29.262	45.506	33.076	26.875	38.788	**34.701**	225, ($p \leq 0.01$)**
	**Bcl_2**	10.541	12.272	13.083	13.049	11.548	**12.099**	-34.7, ($p \leq 0.06$)^{ns}
**PTX+SFN**	**Bax**	58.276	52.356	49.687	73.253	39.698	**54.654**	353.6, ($p \leq 0.0002$)***
	**Bcl_2**	6.5010	9.905	5.455	7.860	5.056	**6.956**	-62.5, ($p \leq 0.007$)**

Data $n = 5$, ($^{*}p \leq 0.05$, $^{**}p \leq 0.01$, $^{***}p \leq 0.001$).
