# Supplementary material for: Optimizing combination therapy in prostate cancer: mechanistic insights into the synergistic effects of Paclitaxel and Sulforaphane-induced apoptosis
Source: BMC Mol Cell Biol. 2024 Mar 4;25:5. doi: 10.1186/s12860-024-00501-z (PMC10910811; doi:10.1186/s12860-024-00501-z)
Supplement: Supplementary file 1 — Supplementary Material 1. [file 12860_2024_501_MOESM1_ESM.zip › Table S2.pdf]

Table S2. presents the densitometric arbitrary scan units of the protein bands adjusted to **GAPDH** for **Bax** and **Bcl2** protein expression in response to **PTX**, **SFN**, or **PTX+SFN** treatments in the **LNCaP** prostate cancer cell line. The statistical analysis performed shows significant increases in **Bax** protein expression and decreases in **Bcl2** protein expression in treated cells compared to non-stimulated control cells (**NS**) and among the mono- and combined treatments. The data further support the notion that **PTX** and **SFN** have additive effects on inducing apoptosis in prostate cancer cells, and the combination treatment may have a synergistic effect.

Treatment	Proteins	Replicates					Mean	% Change relative to control (NS)
		1	2	3	4	5		
**Control (NS)**	**Bax**	23.887	24.286	24.642	17.588	26.354	**23.351**	--
	**Bcl2**	54.386	62.535	49.696	79.053	53.199	**59.774**	--
**PTX**	**Bax**	41.620	40.590	42.333	49.349	28.784	**40.535**	173.5, ($p \leq 0.02$)*
	**Bcl2**	36.924	46.211	25.262	52.320	26.762	**37.496**	-37.2, ($p \leq 0.0005$)***
**SFN**	**Bax**	49.388	44.033	65.5122	43.501	60.101	**52.507**	224.8, ($p \leq 0.003$)**
	**Bcl2**	32.839	33.793	41.586	37.678	32.274	**35.634**	-40.3, ($p \leq 0.01$)**
**PTX+SFN**	**Bax**	50.525	41.535	82.354	58.673	49.436	**56.505**	241.9, ($p \leq 0.01$)**
	**Bcl2**	10.270	6.166	12.411	9.840	12.438	**10.225**	-82.8, ($p \leq 0.001$)***

Data $n = 5$, (*$p \leq 0.05$, **$p \leq 0.01$, ***$p \leq 0.001$).
